# Smoking-Related DNA Methylation is Differentially Associated with Cadmium Concentration in Blood

**DOI:** 10.1007/s10528-020-09965-y

**Published:** 2020-04-28

**Authors:** Jae-Eun Lee, Hye-Ryun Kim, Mee-hee Lee, Nam-Hee Kim, Kyoung-Min Wang, Sang-hyeop Lee, Ok Park, Eun-Jung Hong, Jong-Woo Youn, Young-Youl Kim

**Affiliations:** Division of Biobank for Health Sciences, Center for Genome Science, Korea National Institute of Health, Korea Centers for Disease Control and Prevention, Cheongju, Korea

**Keywords:** Cadmium exposure, DNA methylation, Smoker, Smoking

## Abstract

Tobacco smoking, a risk factor for several human diseases, can lead to alterations in DNA methylation. Smoking is a key source of cadmium exposure; however, there are limited studies examining DNA methylation alterations following smoking-related cadmium exposure. To identify such cadmium exposure-related DNA methylation, we performed genome-wide DNA methylation profiling using DNA samples from 50 smokers and 50 non-smokers. We found that a total of 136 CpG sites (including 70 unique genes) were significantly differentially methylated in smokers as compared to that in non-smokers. The CpG site cg05575921 in the *AHRR* gene was hypomethylated (Δ ß >  − 0.2) in smokers, which was in accordance with previous studies. The rs951295 (within RNA gene LOC105370802) and cg00587941 sites were under-methylated by > 15% in smokers, whereas cg11314779 (within *CELF6*) and cg02126896 were over-methylated by ≥ 15%. We analyzed the association between blood cadmium concentration and DNA methylation level for 50 smokers and 50 non-smokers. DNA methylation rates of 307 CpG sites (including 207 unique genes) were significantly correlated to blood cadmium concentration (linear regression *P* value < 0.001). The four significant loci (cg05575921 and cg23576855 in *AHRR*, cg03636183 in *F2RL3*, and cg21566642) were under-methylated by > 10% in smokers compared to that in non-smokers. In conclusion, our study demonstrated that DNA methylation levels of rs951295, cg00587941, cg11314779, and cg02126896 sites may be new putative indicators of smoking status. Furthermore, we showed that these four loci may be differentially methylated by cadmium exposure due to smoking.

**Electronic Supplementary Material:**

The online version of this article (10.1007/s10528-020-09965-y) contains supplementary material, which is available to authorized users.

## Introduction

Tobacco smoking is a risk factor for various diseases such as cancers, pulmonary and cardiovascular disease, type-2 diabetes, and obesity (Vineis et al. 2004; Mathers and Loncar 2006, Thun et al. 2010, CDC 2008). The World Health Organization (WHO) reported that about 6 million people worldwide die from smoking annually (WHO 2014). Smoking is the primary cause of approximately half-a-million deaths annually in the United States (Mokdad et al. 2004), and of most lung cancer cases (more than 4 of 5 cases) in developed countries (Fairley et al. 2010). Notably, smoking is a main source of cadmium exposure; cadmium accumulates gradually in the human body and has a half-life of 10–30 years (Jarup and Akesson 2009). Cadmium concentrations are much higher in the blood samples of smokers than that in non-smokers (Batariova et al. 2006; El-Agha and Gokmen 2002; Elinder et al. 1983), and smoking-induced increase in urinary cadmium concentration is associated with kidney dysfunction (Mortensen et al. 2011). Cadmium exposure is associated with the development or progression of cancers, cardiovascular dysfunction, nephrotoxicity, and bone damage (Larsson and Wolk 2016; Nordberg et al. 1992; Jarup and Akesson 2009).

Smoking can cause changes in DNA methylation, which plays an essential role in the transcriptional regulation of oncogenes, tumor suppressor genes, and inflammation-related genes (Sundar et al. 2011; Yao and Rahman 2011). Generally, hypermethylation at specific CpG sites in the gene promoter is associated with gene silencing and hypomethylation is related to the activation of gene expression (Yang and Schwartz 2011; Jones 2012). Variation in DNA methylation at specific CpG sites is associated with diseases such as cancers, inflammatory, and pulmonary diseases (Sundar et al. 2011, 2013; Selamat et al. 2012; Morrow et al. 2016; Cheng et al. 2016). Recently, several studies have investigated the changes in DNA methylation associated with smoking, as well as the link between smoking and diseases, such as lung cancer and chronic obstructive pulmonary disease, with respect to DNA methylation status (Ma and Li 2017; Sundar et al. 2017). Cadmium exposure due to smoking can induce alterations in DNA methylation (Virani et al. 2016); however, there are a limited number of studies focused on alterations in DNA methylation due to smoking-related cadmium exposure. In this study, we identified differentially methylated CpG sites in Korean smokers compared to Korean non-smokers, through a microarray-based approach. Further, we also investigated whether these CpG sites were differentially methylated by cadmium exposure due to smoking.

## Methods and Materials

### Study Population

Study participants included 100 non-smokers and 100 current smokers, who enrolled as volunteers for the fifth Korean National Health and Nutrition Examination Survey (2008–2011). We randomly selected subjects based on sex, age, and self-reported smoking history. All selected subjects were male and there was no difference in the average age of the two groups; the average age of the non-smokers was 52 years and that of smokers was 51 years (Table 1). Non-smokers had no smoking history over their lifetime. Smokers had a smoking history of more than 20 cigarettes per day for the past 20 years and first started smoking after the age of 15; the average smoking period per person was 31.54 years and the annual average number of packs of cigarettes per person was 439.64. All study participants provided informed consent.

**Table 1:** Characteristics of study subjects

Variables	Non-smokers (*n* = 100)	Smokers (*n* = 100)
Age, average (SD)	51.89 (9.23)	51.40 (8.32)
Concentration (µg/L) of blood cadmium (SD)	0.83 (4.23)	1.67 (0.68)
Concentration (ng/ml) of urine cotinine (SD)	12.22 (17.16)	1847.95 (1178.87)
Cigarettes per day, average (SD)	–	24.09 (7.77)
Total amount of smoking per person, packs (SD)	–	439.64(141.75)
Smoking years per person, average (SD)	–	31.54 (8.42)

### DNA Methylation Analyses

A total of 50 non-smokers and 50 smokers were randomly selected from participants enrolled in the study for DNA methylation analyses. DNA samples, extracted from buffy coat samples of these subjects, were obtained from the National Biobank of Korea. DNA was bisulfite-converted using EZ DNA Methylation™ Kit (Zymoresearch, California, USA) and DNA methylation profiles were analyzed using the Infinium Human Methylation 450 K BeadChip (Illumina, San Diego, CA), which contains 485,512 CpG sites, according to the manufacturer’s protocol. The methylation rate at each CpG site was calculated by comparing fluorescent signals from methylated and unmethylated sites. The methylation rates are presented as mean beta (ß) values, which ranged from 0 (at a completely unmethylated site) to 1.0 (at a completely methylated site). Delta (Δ) ß value is defined as the difference between the mean ß value of smokers and that of non-smokers (mean ß value of smokers − mean ß value of non-smokers). Methylation rates between the two groups were compared using the independent *t* test statistical method. CpG sites with |Δ ß value|≥ 0.05 and *P* value < 0.01 were considered as differentially methylated.

### Measurement of Urinary Cotinine and Blood Cadmium Levels

Urinary cotinine concentrations were measured by Gas Chromatography Mass Spectrometry (GCMS) using Perkin Elmer Clarus 600 T (PerkinElmer, Finland). Blood cadmium concentrations were measured by Graphite Furnace Atomic Absorption Spectrometry (GFAAS) using PerkinElmer AAnalyst 600 (PerkinElmer, Finland).

### Gene Ontology Analyses

To identify the biological functions of genes that are differentially methylated due to smoking or cadmium exposure, Gene Ontology (biological process terms) analysis was performed using the DAVID Bioinformatics Resources 6.8 Functional Annotation Tool (https://david.ncifcrf.gov/). For this analysis, we used genes differentially methylated by smoking or cadmium exposure. Significant terms were chosen when the Benjamini–Hochberg-corrected *P* value < 0.05.

### Correlation Analyses

The correlation between blood cadmium concentrations and DNA methylation rates was assessed using a linear regression statistical method. For this analysis, the methylation rate (ß value) at each CpG site and blood cadmium concentration (μg/L) from 50 non-smokers and 50 smokers was used. *P* value < 0.001 was considered statistically significant.

## Results

### Comparison of Blood Cadmium and Urinary Cotinine Levels Between Smokers and Non-smokers

The smokers (*n* = 100) and non-smokers (*n* = 100) had mean blood cadmium concentration of 1.67 ± 0.68 μg/L and 0.83 ± 4.23 μg/L, respectively (Table 1). The average concentration of blood cadmium was over 2 times higher in smokers compared to that in non-smokers. The urinary cotinine level is a sensitive biomarker for tobacco smoking (Kulza et al. 2012; Raja et al. 2016). Our results showed that the smokers and non-smokers had mean urinary cotinine concentration of 1847.95 ± 1178.87 ng/mL and 12.22 ± 17.16 ng/mL, respectively. The average concentration of urinary cotinine was over 100 times higher in smokers as compared to that in non-smokers.

### Differential DNA Methylation Between Smokers and Non-smokers

A total of 136 CpG sites, including 70 unique genes, were differentially methylated in smokers compared to non-smokers (|Δ ß value|≥ 0.05; *P* value < 0.01) (Supplementary data 1). Among these, 92 CpG sites, including 51 unique genes, showed under-methylation in smokers compared to non-smokers; 44 CpG sites, including 19 unique genes, exhibited over-methylation in smokers. The average Δ ß value of the 92 under-methylated CpG sites was 0.07 (ranged from − 0.05 to − 0.21), and the average Δ ß value of the 44 over-methylated CpG sites was 0.07 (ranged from 0.05 to 0.15). The top 30 sites with the highest fold change among differentially methylated CpG sites are listed in Table 2. We found 25 under-methylated CpG sites and 5 over-methylated CpG sites in smokers. The cg05575921 site in *AHRR* showed hypomethylation [Δ ß value =  − 0.21; log_2_ (fold change) =  − 0.41]. The rs951295 in RNA gene LOC105370802 [Δ ß value =  − 0.18; log_2_ (fold change) =  − 0.62], cg00587941 [Δ ß value =  − 0.16; log_2_ (fold change) =  − 0.31], and cg23576855 in *AHRR* [Δ ß value =  − 0.17; log_2_ (fold change) =  − 0.40] were under-methylated, while cg11314779 in *CELE6* [Δ ß value = 0.15; log_2_ (fold change) = 0.38] and cg02126896 [Δ ß value = 0.15; log_2_ (fold change) = 0.39] were over-methylated.

**Table 2:** Top 30 differentially methylated CpG sites in non-smokers (*n* = 50) and smokers (*n* = 50)

Probe ID	Gene symbol	β value of non-smokers	β value of smokers	Δ β value	*t* test *P* value	Log_2_ (fold change)^a^
rs951295	LOC105370802	0.51	0.33	− 0.18	0.002	− 0.62
cg02249911	CASP3;CCDC111	0.24	0.17	− 0.07	0.005	− 0.52
cg06644428		0.19	0.13	− 0.06	< 0.001	− 0.51
cg20059012	RARG	0.18	0.13	− 0.05	< 0.001	− 0.51
cg25538415	DCAKD	0.24	0.17	− 0.07	0.006	− 0.47
cg26374206	ZNF709	0.18	0.14	− 0.05	< 0.001	− 0.43
cg00909514	C1orf106	0.21	0.15	− 0.05	< 0.001	− 0.43
cg05575921	AHRR	0.83	0.62	− 0.21	< 0.001	− 0.41
cg23576855	AHRR	0.71	0.54	− 0.17	< 0.001	− 0.40
cg19211853	RBM26	0.26	0.20	− 0.06	< 0.001	− 0.40
cg21566642		0.52	0.41	− 0.12	< 0.001	− 0.37
cg07339236	ATP9A	0.23	0.18	− 0.05	< 0.001	− 0.37
cg14743534	RASA4CP;FLJ35390	0.22	0.17	− 0.05	0.004	− 0.36
cg09682128		0.22	0.17	− 0.05	0.006	− 0.34
cg17246140	HAAO	0.22	0.17	− 0.05	< 0.001	− 0.34
cg14817490	AHRR	0.32	0.26	− 0.07	< 0.001	− 0.33
cg19980771	SLC22A16	0.33	0.26	− 0.07	0.006	− 0.33
cg12075498	JAK1	0.31	0.25	− 0.06	0.002	− 0.33
cg05951221		0.43	0.34	− 0.08	< 0.001	− 0.31
cg19080354	ATHL1	0.40	0.32	− 0.08	0.008	− 0.31
cg21974656		0.39	0.32	− 0.08	0.002	− 0.31
cg00587941		0.85	0.69	− 0.16	0.001	− 0.31
cg17759274	LGALS7	0.30	0.24	− 0.06	0.005	− 0.30
cg12339131		0.71	0.58	− 0.13	0.003	− 0.30
cg25327888	CACNB2	0.25	0.20	− 0.05	0.005	− 0.29
cg11314779	CELF6	0.50	0.65	0.15	0.005	0.38
cg02126896		0.48	0.63	0.15	0.006	0.39
cg01134012	GSDMD	0.13	0.18	0.05	0.002	0.52
cg14721632	GSDMD	0.14	0.23	0.08	0.007	0.66
cg05419812		0.11	0.18	0.07	0.005	0.68

^a^Log_2_ (β value of smokers/β value of non − smokers)

We performed gene ontology analysis on 70 genes that were differentially methylated in smokers (Table 3). *AHRR* (cg05575921, cg23576855, cg14817490, cg03991871, cg21161138, cg25648203), *GFI1* (cg09935388), *HOPX* (cg25456368), *RARA* (cg19572487), *RARG* (cg20059012), *REST* (cg25313468), and *ZFP57* (cg12463578) are associated with negative regulation of transcription, and all were under-methylated in smokers.

**Table 3:** Gene ontology analysis of 70 differentially methylated genes in non-smokers and smokers

Gene ontology accession	Gene ontology term	Genes	Count	P-value
GO:0002068	Glandular epithelial cell development	RARG, RARA	2	0.01
GO:0060534	Trachea cartilage development	RARG, RARA	2	0.01
GO:0045955	Negative regulation of calcium ion-dependent exocytosis	ADRA2A, REST	2	0.02
GO:0000122	Negative regulation of transcription from RNA polymerase II promoter	AHRR, RARG, ZFP57, HOPX, RARA, GFI1, REST	7	0.03
GO:0031076	Embryonic camera-type eye development	RARG, RARA	2	0.04
GO:0031641	Regulation of myelination	RARG, RARA	2	0.04

### DNA Methylation Associated with Cadmium Exposure

To identify cadmium exposure-related DNA methylation, we evaluated the correlation between blood cadmium concentration and DNA methylation rate at each CpG site, using genome-wide DNA methylation data obtained from 50 smokers and 50 non-smokers. The results showed that DNA methylation rates at 307 CpG sites, including 207 unique genes, were significantly correlated to the blood cadmium concentrations of the study subjects (*P* value < 0.001) (data not shown). The top ten sites with the most significant correlations were cg03991871, cg05575921, cg12806681, cg21161138, and cg23576855 in *AHRR*, cg03636183 in *F2RL3*, cg05951221, cg01940273, cg19859270 in *GPR15*, and cg21566642. We analyzed the biological functions of 207 genes (Table 4); these genes, including *AHRR*, *F2RL3*, *HOPX*, *RARA*, and *RARB,* were found to be typically associated with transcription regulation and signal transduction.

**Table 4:** Gene ontology analysis of genes showing significant correlation between DNA methylation rate and blood cadmium concentration

Gene ontology accession	Gene ontology term	Genes	Count	P-value
GO:0007165	Signal transduction	F2RL3, RTKN, ELK3, GNG12, CCL22, UNC5B, DGKD, GATA3, RASGRP1, ADRA2A, RARA, PITPNC1, RARB, PDE8A, ITPK1, RET, STK24, CACNA1I, PI4KB, SFRP5, CD38, ARRB1, TNFRSF10D, REM2, HIVEP3, CD79B	26	< 0.01
GO:0000122	Negative regulation of transcription from RNA polymerase II promoter	DNMT3A, KLF16, RYBP, WWC2, TMPRSS6, PPP1R13L, SIRT2, AHRR, SIN3B, PCBP3, ETS2, GATA3, WWC3, HOPX, RARA, GFI1, RARB, ETV6, NFIC	19	< 0.01
GO:0045907	Positive regulation of vasoconstriction	AVPR2, CD38, AVPR1B, PTAFR	4	< 0.01
GO:0030168	Platelet activation	F2RL3, DGKD, ARRB1, GNA12, ADRA2A, PIK3R5	6	0.01
GO:0031641	Regulation of myelination	RARA, RARB, SIRT2	3	0.01
GO:0001934	Positive regulation of protein phosphorylation	MOB2, OPRL1, ARRB1, RASGRP1, FLOT1, PDE8A	6	0.01
GO:0030036	Actin cytoskeleton organization	MTSS1, TNXB, MOB2, SPTBN5, ADRA2A, ARHGEF17	6	0.01
GO:0032147	Activation of protein kinase activity	SLC11A1, PRKAG2, TGFBR2, ADRA2A	4	0.01
GO:0045944	Positive regulation of transcription from RNA polymerase II promoter	MAFG, ARID3B, ELK3, TMPRSS6, SIRT2, CBFB, RGMA, MEF2D, SLC11A1, AHRR, ARRB1, ETS2, GATA3, RARA, NFE2L2, RARB, ETV6, NFIC, LRP5	19	0.01
GO:0032355	Response to estradiol	DNMT3A, CD38, OPRL1, RARA, NQO1	5	0.01
GO:0032753	Positive regulation of interleukin-4 production	GATA3, RARA, LGALS9	3	0.02
GO:0009609	Response to symbiotic bacterium	GPX1, PTAFR	2	0.02
GO:0003148	Outflow tract septum morphogenesis	TGFBR2, RARA, RARB	3	0.02
GO:0007411	Axon guidance	SPTBN5, GATA3, PTPRA, SEMA3A, BOC, SPTB	6	0.02
GO:0001819	Positive regulation of cytokine production	SLC11A1, FLOT1, ADRA2A	3	0.02
GO:0030099	Myeloid cell differentiation	GFI1, PRKX, CBFB	3	0.02
GO:0030534	Adult behavior	MAFG, CNTNAP2, SHANK2	3	0.03
GO:1904381	Golgi apparatus mannose trimming	MAN1A1, MAN1C1	2	0.03
GO:0002068	Glandular epithelial cell development	RARA, RARB	2	0.03
GO:0014065	Phosphatidylinositol 3-kinase signaling	GATA3, PIK3R5, SIRT2	3	0.03
GO:0032689	Negative regulation of interferon-gamma production	GATA3, RARA, LGALS9	3	0.03
GO:0006366	Transcription from RNA polymerase II promoter	MAFG, MEF2D, ARRB1, GATA3, HIVEP3, ARID3B, ELK3, NFE2L2, ETV6, NFIC, CBFB	11	0.04
GO:0010332	Response to gamma radiation	GPX1, GATA3, BRCA2	3	0.04
GO:0007030	Golgi organization	SYNE1, RAB43, SPTBN5, ATL3	4	0.04
GO:0035799	Ureter maturation	RET, GATA3	2	0.04
GO:0043415	Positive regulation of skeletal muscle tissue regeneration	TGFBR2, HOPX	2	0.04
GO:1904058	Positive regulation of sensory perception of pain	OPRL1, PTAFR	2	0.04
GO:0007596	Blood coagulation	MAFG, F2RL3, GATA3, PRKAR1B, GNA12, ITPK1	6	0.04
GO:0003091	Renal water homeostasis	AVPR2, PRKAR1B, AQP1	3	0.04
GO:0002088	Lens development in camera-type eye	GATA3, TGFBR2, LIM2	3	0.04

### Identification of Genes Commonly Associated with Smoking and Blood Cadmium Exposure

To identify DNA methylation induced by cadmium exposure due to smoking, we selected CpG sites that were differentially methylated in smokers from among the cadmium exposure-associated DNA methylation described above. Thirty-eight CpG sites (including 23 unique genes) were identified (Table 5). The cg05575921 and cg23576855 in *AHRR*, cg03636183 in *F2RL3*, and cg21566642, showed a Δ ß value <  − 1.0 for DNA methylation rates between smokers and non-smokers.

**Table 5:** Thirty-eight CpG sites that were differentially methylated due to smoking and blood cadmium concentration

Probe	Gene symbol	β value of non-smokers	β value of smokers	Δ β value	*t* test P value	Log_2_ (fold change)^a^
cg06644428		0.19	0.13	− 0.06	< 0.001	− 0.51
cg00909514	C1orf106	0.21	0.15	− 0.05	< 0.001	− 0.43
cg05575921	AHRR	0.83	0.62	− 0.21	< 0.001	− 0.41
cg23576855	AHRR	0.71	0.54	− 0.17	< 0.001	− 0.40
cg19211853	RBM26	0.26	0.20	− 0.06	< 0.001	− 0.40
cg21566642		0.52	0.41	− 0.12	< 0.001	− 0.37
cg07339236	ATP9A	0.23	0.18	− 0.05	< 0.001	− 0.37
cg14817490	AHRR	0.32	0.26	− 0.07	< 0.001	− 0.33
cg17246140	HAAO	0.22	0.17	− 0.05	< 0.001	− 0.34
cg05951221		0.43	0.34	− 0.08	< 0.001	− 0.31
cg26827373	ZNF844	0.27	0.22	− 0.05	< 0.001	− 0.28
cg03636183	F2RL3	0.69	0.58	− 0.12	< 0.001	− 0.26
cg07381806	MOB3A	0.48	0.41	− 0.07	< 0.001	− 0.23
cg25189904	GNG12	0.48	0.41	− 0.07	< 0.001	− 0.23
cg23161492	ANPEP	0.38	0.32	− 0.05	< 0.001	− 0.22
cg13184736	GNG12	0.44	0.38	− 0.05	< 0.001	− 0.19
cg06126421		0.74	0.65	− 0.09	< 0.001	− 0.19
cg21140898		0.38	0.33	− 0.05	< 0.001	− 0.19
cg01940273		0.60	0.53	− 0.07	< 0.001	− 0.18
cg19717773	GNA12	0.72	0.65	− 0.07	< 0.001	− 0.16
cg01208318		0.52	0.48	− 0.05	< 0.001	− 0.14
cg05329352	ADRA2A	0.61	0.55	− 0.06	< 0.001	− 0.14
cg19572487	RARA	0.55	0.50	− 0.05	< 0.001	− 0.14
cg26889659	EXOC2	0.77	0.70	− 0.07	< 0.001	− 0.13
cg18446336	GNA12	0.58	0.52	− 0.05	< 0.001	− 0.14
cg20698421	SLC1A4	0.61	0.56	− 0.05	< 0.001	− 0.13
cg21161138	AHRR	0.70	0.64	− 0.06	< 0.001	− 0.12
cg16836311	MAN1C1	0.64	0.59	− 0.05	< 0.001	− 0.12
cg09069072	TMEM51	0.81	0.75	− 0.06	< 0.001	− 0.11
cg03991871	AHRR	0.84	0.78	− 0.06	< 0.001	− 0.11
cg09935388	GFI1	0.77	0.72	− 0.05	< 0.001	− 0.10
cg26361535	ZC3H3	0.74	0.69	− 0.05	< 0.001	− 0.10
cg25648203	AHRR	0.76	0.72	− 0.05	< 0.001	− 0.09
cg12803068	MYO1G	0.76	0.82	0.06	< 0.001	0.12
cg19758448	PGAP3	0.53	0.58	0.05	< 0.001	0.12
cg05059607	PITPNC1	0.45	0.49	0.05	< 0.001	0.14
cg08035323		0.30	0.35	0.05	< 0.001	0.21
cg12423733	MAS1L	0.23	0.28	0.05	< 0.001	0.26

^a^Log_2_ (β value of smokers/β value of non-smokers)

## Discussion

In this study, we identified smoking-induced methylation alterations at 136 CpG sites (including 70 unique genes). The cg05575921 site in *AHRR* showed hypomethylation in smokers, in accordance with previous studies (Zeilinger et al. 2013; Dogan et al. 2014; Lee et al. 2017). These data support the suggestion that methylation levels of *AHRR* (cg05575921) may be used as an indicator of smoking intensity (Beach et al. 2015). Our findings were consistent with those of previous studies on smoking-associated DNA methylation at other CpG sites as well. Studies have reported that cg21161138 and cg26703534 in *AHRR*, cg01940273, cg06126421, cg21566642 (Zeilinger et al. 2013; Dogan et al. 2014), cg03636183 in *F2RL3* (Zeilinger et al. 2013; Breitling et al. 2011), and cg19572487 in *RARA* (Zeilinger et al. 2013) are under-methylated in smokers. These studies included participants from the KORA S4 survey, African American females from the states of Iowa and Georgia, and general population-based epidemiological ESTHER study participants (Zeilinger et al. 2013; Dogan et al. 2014; Breitling et al. 2011). The combined data revealed that the DNA methylation patterns of 8 CpG sites (cg05575921, cg21161138, and cg26703534 in *AHRR*, cg01940273, cg06126421, cg21566642, cg03636183 in *F2RL3*, and cg19572487 in *RARA*) might change depending on the smoking status, regardless of race. In addition, we identified that rs951295, cg00587941, cg11314779, and cg02126896 were under- or over-methylated by ≥ 15% in smokers. These results indicate that methylation of these 4 CpG sites may be new candidate indicators for long-term smoking exposure. The biological implications of methylation changes at rs951295 (within RNA gene LOC105370802), cg00587941, and cg02126896 remain unknown. The cg11314779 site is located in the intron of *CELF6*. Since the *CELF6* is associated with addiction (Bryant and Yazdani 2016), it may be interesting to study the relationship between methylation of cg11314779 and addiction.

Smoking is the main source of cadmium exposure. The blood cadmium concentrations of smokers were found to be significantly higher than those of non-smokers in this study. We identified cadmium exposure-related DNA methylation of 307 CpG sites (including 207 unique genes). *CCL22*, a signal transduction-related gene, was differentially methylated by cadmium exposure. A previous study on the relationship between cadmium exposure and *CCL22* was retrieved from the PubMed database (February 17, 2020). The mRNA level of *CCL22* decreased in antigen-activated lymphocytes due to cadmium treatment (Ebaid et al. 2014). Further studies are needed to determine whether DNA methylation of *CCL22* due to cadmium exposure affects the gene expression. cg05575921 and cg23576855 in *AHRR*, cg03636183 in *F2RL3*, and cg21566642 that have not been previously reported to be associated with cadmium exposure were included in our study. These were under-methylated by > 10% in smokers compared to that in non-smokers. DNA methylation of cg05575921 in *AHRR* (Beach et al. 2015) and cg03636183 in *F2RL3* (Zeilinger et al. 2013; Breitling et al. 2011) is the putative indicator for smoking. Taken together, these CpG sites (cg05575921 and cg23576855 in *AHRR*, cg03636183 in *F2RL3*, and cg21566642) may be differentially methylated by cadmium exposure due to smoking.

The Gene Ontology terms were analyzed to identify the biological functions of genes differentially methylated due to smoking and cadmium exposure. Among the 70 genes found to be differentially methylated in smokers compared to non-smokers, *HOPX* (cg25456368), *RARG* (cg20059012), and *ZFP57* (cg12463578) genes, found to be under-methylated in the non-promoter regions, are involved in negative transcription regulation. Previous studies have reported that hyper-methylation in non-promoter regions of *MMP9* (Falzone et al. 2016) and *CDKN2A* (Ben-Dayan et al. 2017) was associated with transcriptional activation. Therefore, the expression of *HOPX*, *RARG*, and *ZFP57* genes might also be regulated by DNA methylation in the non-promoter regions. Glandular epithelial cell development (*RARA*), negative regulation of transcription from RNA polymerase II promoter (*AHRR*), and regulation of myelination (*RARA*) are the biological functions of some genes that were differentially methylated by both smoking and cadmium exposure. The *RARA* gene acts as retinoic acid receptor, nuclear receptor, and steroid hormone receptor (Tsaprouni et al. 2014). Recently, it has been reported that differential DNA methylation in *RARA* is associated with smoking in African Americans (Barcelona et al. 2019). Our data showed that cg19572487 in *RARA* was under-methylated by 9% (Δ ß-value =  − 0.05) in smokers compared to that in non-smokers (data not shown). Thus, alterations to DNA methylation in *RARA* can be caused by cadmium exposure as well as smoking.

The urinary cotinine level is a sensitive biomarker for tobacco smoking (Kulza et al. 2012; Raja et al. 2016) Behera et al. (2003). showed that mean urinary cotinine levels were 2736.20 ± 983.29 ng/mL and 7.30 ± 2.47 ng/mL in smokers and non-smokers, respectively. Sharma et al. (2019) reported that mean urinary cotinine levels were 1043.69 ± 1514.01 ng/mL and 13.60 ± 12.73 ng/mL in smokers and non-smokers, respectively. Our study found that smokers had a mean urinary cotinine concentration of 1847.95 ± 1178.87 ng/mL and non-smokers had a mean of 12.22 ± 17.16 ng/mL. Thus, these findings suggest that urinary cotinine values may be an indicator for smoking.

In conclusion, our study showed that 136 CpG sites (including 70 unique genes) were differentially methylated by smoking. Among these, DNA methylation levels of rs951295, cg00587941, cg11314779, and cg02126896 sites may be new putative indicators for smoking intensity. Furthermore, DNA methylation at cg05575921 and cg23576855 in *AHRR*, cg03636183 in *F2RL3*, and cg21566642 may be altered by smoking-induced cadmium exposure. These findings provide a novel insight into smoking-induced genetic alterations that might be involved in associated diseases.

## Electronic Supplementary Material

Below is the link to the electronic supplementary material.

Supplementary file 1 (DOCX 47 kb)
