# Supplementary material for: Smoking-Related DNA Methylation is Differentially Associated with Cadmium Concentration in Blood
Source: Biochem Genet. 2020 Apr 28;58(4):617–30. doi: 10.1007/s10528-020-09965-y (PMC7378121; doi:10.1007/s10528-020-09965-y)
Supplement: Supplementary file 1 — (DOCX 47 kb) [file 10528_2020_9965_MOESM1_ESM.docx]

Supplementary Data 1 Differentially methylated CpG sites in non-smokers and smokers

| **Probe ID** | **Gene symbol** | **β-value of**  **non-smokers** | **β-value of**  **smokers** | **Δ β-value** | ***t* test**  **P-value** |
| --- | --- | --- | --- | --- | --- |
| cg05575921 | AHRR | 0.83 | 0.62 | -0.21 | < 0.001 |
| rs951295 | LOC105370802 | 0.51 | 0.33 | -0.18 | 0.002 |
| cg23576855 | AHRR | 0.71 | 0.54 | -0.17 | < 0.001 |
| cg00587941 |  | 0.85 | 0.69 | -0.16 | 0.001 |
| cg12339131 |  | 0.71 | 0.58 | -0.13 | 0.003 |
| cg21566642 |  | 0.52 | 0.41 | -0.12 | < 0.001 |
| cg03636183 | F2RL3 | 0.69 | 0.58 | -0.12 | < 0.001 |
| cg02775404 |  | 0.76 | 0.65 | -0.11 | 0.007 |
| cg01153376 | MSLN;MIR662 | 0.84 | 0.73 | -0.11 | 0.008 |
| cg24320034 | PRTFDC1 | 0.84 | 0.74 | -0.10 | 0.004 |
| cg06126421 |  | 0.74 | 0.65 | -0.09 | < 0.001 |
| cg14533999 | ADAMTS2 | 0.89 | 0.81 | -0.08 | 0.008 |
| cg05951221 |  | 0.43 | 0.34 | -0.08 | < 0.001 |
| cg19080354 | ATHL1 | 0.40 | 0.32 | -0.08 | 0.008 |
| cg21974656 |  | 0.39 | 0.32 | -0.08 | 0.002 |
| cg19717773 | GNA12 | 0.72 | 0.65 | -0.07 | < 0.001 |
| cg02249911 | CASP3;CCDC111 | 0.24 | 0.17 | -0.07 | 0.005 |
| cg25189904 | GNG12;GNG12-AS1 | 0.48 | 0.41 | -0.07 | < 0.001 |
| cg19851563 | GNG7 | 0.68 | 0.61 | -0.07 | 0.003 |
| cg07381806 | MOB3A | 0.48 | 0.41 | -0.07 | < 0.001 |
| cg01940273 |  | 0.60 | 0.53 | -0.07 | < 0.001 |
| cg09658497 | GNA12 | 0.78 | 0.71 | -0.07 | < 0.001 |
| cg26889659 | EXOC2 | 0.77 | 0.70 | -0.07 | < 0.001 |
| cg25538415 | DCAKD | 0.24 | 0.17 | -0.07 | 0.006 |
| cg14817490 | AHRR | 0.32 | 0.26 | -0.07 | < 0.001 |
| cg19977566 | CACNB2 | 0.74 | 0.67 | -0.07 | 0.005 |
| cg19980771 | SLC22A16 | 0.33 | 0.26 | -0.07 | 0.006 |
| cg26748578 | SLC1A4 | 0.41 | 0.35 | -0.07 | 0.001 |
| cg06611532 |  | 0.76 | 0.70 | -0.06 | 0.005 |
| cg12075498 | JAK1 | 0.31 | 0.25 | -0.06 | 0.002 |
| cg19211853 | RBM26 | 0.26 | 0.20 | -0.06 | < 0.001 |
| cg03991871 | AHRR | 0.84 | 0.78 | -0.06 | < 0.001 |
| cg10663765 |  | 0.69 | 0.63 | -0.06 | < 0.001 |
| cg00876757 | RNU6-76 | 0.61 | 0.55 | -0.06 | 0.003 |
| cg09069072 | TMEM51 | 0.81 | 0.75 | -0.06 | < 0.001 |
| cg03708092 | LPAL2 | 0.78 | 0.72 | -0.06 | 0.002 |
| cg21161138 | AHRR | 0.70 | 0.64 | -0.06 | < 0.001 |
| cg10225865 |  | 0.52 | 0.46 | -0.06 | 0.007 |
| cg03996861 |  | 0.56 | 0.50 | -0.06 | 0.001 |
| cg05329352 | ADRA2A | 0.61 | 0.55 | -0.06 | < 0.001 |
| cg21869609 | LINGO3 | 0.81 | 0.75 | -0.06 | 0.002 |
| cg17759274 | LGALS7 | 0.30 | 0.24 | -0.06 | 0.005 |
| cg06644428 |  | 0.19 | 0.13 | -0.06 | < 0.001 |
| cg25313468 | REST | 0.63 | 0.58 | -0.06 | < 0.001 |
| cg14716990 |  | 0.53 | 0.48 | -0.05 | 0.003 |
| cg05696877 | IFI44L | 0.63 | 0.58 | -0.05 | 0.006 |
| cg13184736 | GNG12;GNG12-AS1 | 0.44 | 0.38 | -0.05 | < 0.001 |
| cg20219891 |  | 0.67 | 0.62 | -0.05 | 0.002 |
| cg23161492 | ANPEP | 0.38 | 0.32 | -0.05 | < 0.001 |
| cg19078576 | LOC285696;BASP1 | 0.31 | 0.26 | -0.05 | 0.001 |
| cg20059012 | RARG | 0.18 | 0.13 | -0.05 | < 0.001 |
| cg21733098 |  | 0.68 | 0.63 | -0.05 | < 0.001 |
| cg00909514 | C1orf106 | 0.21 | 0.15 | -0.05 | < 0.001 |
| cg17179862 | HOXB-AS3;HOXB6 | 0.68 | 0.63 | -0.05 | 0.001 |
| cg07339236 | ATP9A | 0.23 | 0.18 | -0.05 | < 0.001 |
| cg21816330 | RAB34;NARR | 0.32 | 0.27 | -0.05 | 0.004 |
| cg10906729 | HOXB-AS3;HOXB6 | 0.75 | 0.70 | -0.05 | 0.004 |
| cg18446336 | GNA12 | 0.58 | 0.52 | -0.05 | < 0.001 |
| cg20698421 | SLC1A4 | 0.61 | 0.56 | -0.05 | < 0.001 |
| cg22900607 | GNG7 | 0.71 | 0.65 | -0.05 | 0.002 |
| cg09935388 | GFI1 | 0.77 | 0.72 | -0.05 | < 0.001 |
| cg21587238 |  | 0.72 | 0.67 | -0.05 | 0.006 |
| cg16836311 | MAN1C1 | 0.64 | 0.59 | -0.05 | < 0.001 |
| cg03803541 | HOXB-AS3;HOXB6 | 0.67 | 0.62 | -0.05 | 0.001 |
| cg02583091 | SLC39A4 | 0.32 | 0.27 | -0.05 | 0.004 |
| cg17316649 | HLA-DRB1 | 0.83 | 0.78 | -0.05 | 0.008 |
| cg19572487 | RARA | 0.55 | 0.50 | -0.05 | < 0.001 |
| cg18835078 | PCDHGA1 | 0.78 | 0.73 | -0.05 | 0.002 |
| cg01208318 |  | 0.52 | 0.48 | -0.05 | < 0.001 |
| cg12289251 | CACNB2 | 0.40 | 0.36 | -0.05 | 0.006 |
| cg23615741 | NKX2-3 | 0.56 | 0.51 | -0.05 | 0.001 |
| cg19220282 | SLC1A4 | 0.45 | 0.40 | -0.05 | < 0.001 |
| cg14015656 | LOC100499484-C9ORF174;C9orf174 | 0.84 | 0.79 | -0.05 | < 0.001 |
| cg26827373 | ZNF844 | 0.27 | 0.22 | -0.05 | < 0.001 |
| cg14743534 | RASA4CP;FLJ35390 | 0.22 | 0.17 | -0.05 | 0.004 |
| cg26361535 | ZC3H3 | 0.74 | 0.69 | -0.05 | < 0.001 |
| cg01294327 | LINGO3 | 0.70 | 0.66 | -0.05 | < 0.001 |
| cg02880119 |  | 0.66 | 0.61 | -0.05 | 0.001 |
| cg12924095 | G3BP1 | 0.52 | 0.47 | -0.05 | < 0.001 |
| cg05505103 |  | 0.64 | 0.59 | -0.05 | 0.002 |
| cg26374206 | ZNF709 | 0.18 | 0.14 | -0.05 | < 0.001 |
| cg03165014 | ITGBL1 | 0.31 | 0.26 | -0.05 | 0.002 |
| cg25456368 | HOPX | 0.54 | 0.49 | -0.05 | 0.003 |
| cg09918751 | ADAMTS17 | 0.80 | 0.75 | -0.05 | 0.004 |
| cg25648203 | AHRR | 0.76 | 0.72 | -0.05 | < 0.001 |
| cg21140898 |  | 0.38 | 0.33 | -0.05 | < 0.001 |
| cg09682128 |  | 0.22 | 0.17 | -0.05 | 0.006 |
| cg10181414 | GNG7 | 0.68 | 0.63 | -0.05 | 0.004 |
| cg12463578 | ZFP57 | 0.58 | 0.54 | -0.05 | 0.006 |
| cg25327888 | CACNB2 | 0.25 | 0.20 | -0.05 | 0.005 |
| cg11324650 |  | 0.57 | 0.52 | -0.05 | 0.001 |
| cg17246140 | HAAO | 0.22 | 0.17 | -0.05 | < 0.001 |
| cg05718076 |  | 0.52 | 0.57 | 0.05 | 0.002 |
| cg18933685 | SDK1 | 0.32 | 0.37 | 0.05 | < 0.001 |
| cg15542713 | HIVEP3 | 0.50 | 0.55 | 0.05 | 0.001 |
| cg00591234 | HIST1H3C | 0.81 | 0.86 | 0.05 | 0.007 |
| cg19758448 | PGAP3 | 0.53 | 0.58 | 0.05 | < 0.001 |
| cg08173263 | LOC100507373;LPHN1 | 0.41 | 0.46 | 0.05 | < 0.001 |
| cg05059607 | PITPNC1 | 0.45 | 0.49 | 0.05 | < 0.001 |
| cg12423733 | MAS1L | 0.23 | 0.28 | 0.05 | < 0.001 |
| cg08035323 |  | 0.30 | 0.35 | 0.05 | < 0.001 |
| cg07243930 | TRPC2 | 0.73 | 0.78 | 0.05 | < 0.001 |
| cg27202779 | CARD11 | 0.30 | 0.35 | 0.05 | 0.005 |
| cg22132788 | MYO1G | 0.88 | 0.93 | 0.05 | < 0.001 |
| cg17291887 |  | 0.58 | 0.64 | 0.05 | 0.003 |
| cg24249791 |  | 0.52 | 0.57 | 0.05 | 0.002 |
| cg23613051 | SH3BP2 | 0.28 | 0.33 | 0.05 | 0.001 |
| cg01134012 | GSDMD | 0.13 | 0.18 | 0.05 | 0.002 |
| cg22078781 |  | 0.29 | 0.34 | 0.05 | 0.002 |
| cg26678138 |  | 0.36 | 0.41 | 0.06 | 0.001 |
| cg24049493 | HIVEP3 | 0.25 | 0.31 | 0.06 | < 0.001 |
| cg08721802 |  | 0.37 | 0.42 | 0.06 | 0.003 |
| cg15693572 |  | 0.57 | 0.62 | 0.06 | < 0.001 |
| cg00600477 |  | 0.46 | 0.52 | 0.06 | 0.004 |
| cg16274205 |  | 0.42 | 0.48 | 0.06 | 0.005 |
| cg12803068 | MYO1G | 0.76 | 0.82 | 0.06 | < 0.001 |
| cg02716490 |  | 0.73 | 0.80 | 0.07 | 0.002 |
| cg05419812 |  | 0.11 | 0.18 | 0.07 | 0.005 |
| cg24441899 | SDK1 | 0.46 | 0.52 | 0.07 | 0.002 |
| cg23480021 |  | 0.62 | 0.69 | 0.07 | 0.002 |
| cg05948955 | GALNT9 | 0.66 | 0.73 | 0.07 | 0.009 |
| cg01334504 | U2AF1 | 0.79 | 0.86 | 0.07 | 0.001 |
| cg03274391 |  | 0.55 | 0.63 | 0.07 | 0.001 |
| cg00762069 | COL6A4P2 | 0.70 | 0.78 | 0.08 | 0.002 |
| cg14721632 | GSDMD | 0.14 | 0.23 | 0.08 | 0.007 |
| cg22953237 |  | 0.72 | 0.81 | 0.09 | 0.003 |
| cg25120325 | PNLIPRP2 | 0.52 | 0.60 | 0.09 | 0.003 |
| cg10858640 | SDK1 | 0.72 | 0.82 | 0.09 | 0.003 |
| cg07629625 | JAKMIP3 | 0.53 | 0.63 | 0.10 | 0.006 |
| cg02193806 | ACSM3 | 0.63 | 0.73 | 0.10 | 0.008 |
| cg12036633 |  | 0.75 | 0.86 | 0.11 | 0.004 |
| cg18828268 | BRIP1 | 0.65 | 0.76 | 0.11 | 0.009 |
| cg13474262 |  | 0.63 | 0.75 | 0.12 | 0.001 |
| rs7746156 |  | 0.67 | 0.81 | 0.14 | 0.008 |
| cg02126896 |  | 0.48 | 0.63 | 0.15 | 0.006 |
| cg11314779 | CELF6 | 0.50 | 0.65 | 0.15 | 0.005 |
